# Multiprofessioneller Behandlungsansatz bei chronischen Rückenschmerzen

**DOI:** 10.1007/s00393-022-01258-6

**Published:** 2022-09-02

**Authors:** Tobias Manigold, Brigitte E. Gantschnig, Konrad Streitberger

**Affiliations:** 1 Universitätsklinik für Rheumatologie und Immunologie, Inselspital, Universitätsspital Bern, Universität Bern, Freiburgstr. 16p, 3010 Bern, Schweiz; 2 Institut für Ergotherapie, Departement Gesundheit, ZHAW Zürcher Hochschule für Angewandte Wissenschaften, Zürich, Schweiz; 3 Universitätsklinik für Anästhesiologie und Schmerztherapie, Inselspital, Universitätsspital Bern, Universität Bern, Bern, Schweiz

**Keywords:** Interprofessionelle Rehabilitation, Interdisziplinäre Schmerzsprechstunde, Multimodale Schmerztherapie, Biopsychosoziales Säulenmodell, Multiprofessionelle Rehabilitationsprogramme, Interprofessional rehabilitation, Interdisciplinary pain consultation, Multimodal pain therapy, Biopsychosocial pillar model, Multiprofessional rehabilitation program

## Abstract

Internationale Leitlinien empfehlen bei anhaltenden Rückenschmerzen bereits frühzeitig die Einbindung verschiedener Professionen und Disziplinen. Damit in Verbindung werden häufig Begriffe wie multiprofessionelle oder interprofessionelle Therapieansätze genannt ohne eine einheitliche Vorstellung, was darunter verstanden wird. Der vorliegende Beitrag soll Orientierung geben, welche multiprofessionellen Therapieansätze es bei chronischen Rückenschmerzen gibt und wie diese in ein interdisziplinäres und interprofessionelles multimodales Therapiekonzept integriert werden können. Dies stellen wir in einem biopsychosozialen Säulenmodell dar, das für jeden Patienten individuell erstellt werden sollte.

## Abstract (en)

International guidelines recommend involving various professions and disciplines at an early stage in the event of chronic back pain. In connection with this, terms such as multiprofessional or interprofessional interventions are often mentioned without a uniform idea of what they mean. This article is intended to provide an overview of multiprofessional interventions for patients with chronic back pain and the integration into a meaningful interdisciplinary and interprofessional multimodal treatment concept. This is illustrated in a biopsychosocial pillar model, which should be pursued for each patient individually.

## Epidemiologie

Rückenschmerz gilt weltweit als führende Ursache für Produktivitätsverlust und gelebte Jahre mit Behinderung [9]. Chronischer Rückenschmerz ist einer der häufigsten Gründe für hausärztliche Konsultationen. In Deutschland ergab eine Erhebung im Jahr 2020, dass 61,3 % der Befragten im vergangenen Jahr über akute und 15,5 % über chronische Rückenschmerzen berichten [14]. Gemäß Schweizer „Rückenreport 2020“ hatten 88 % der Bevölkerung 1‑mal im Leben und 17 % der Bevölkerung wöchentlich Rückenschmerzen [16]. In den meisten Studien ist das Vorkommen in Ländern mit höherem Durchschnittseinkommen und bei Männern erhöht. Die Inzidenz ist in Ländern mit höherem Durchschnittseinkommen, bei Frauen und Personen mit einem schwächeren sozioökonomischen Hintergrund erhöht [5]; 27 % der Befragten sind schon mindestens 1‑mal bei der Arbeit aufgrund von Rückenschmerzen ausgefallen [5]. Patienten mit chronischen Rückenschmerzen leiden bisweilen im Alltag stark und sind in ihren Aktivitäten und der gesellschaftlichen Partizipation beeinträchtigt. Dadurch werden enorme direkte und indirekte Kosten verursacht: Diese belaufen sich zu einem Drittel auf die Behandlung und zu zwei Dritteln auf Produktivitätsausfälle und -minderung. Letztere machen in der Schweiz 7460 Mio. Franken pro Jahr aus [25] und verdeutlichen den Bedarf an interprofessionellen Behandlungen.

## Präsentation, Anamnese und Untersuchung

Bei der Erstvorstellung des Patienten sind eine gründliche ärztliche Anamnese und Untersuchung essenziell, die die Weichen für weitere Untersuchungen und die Behandlung stellt.

Erstens sollten bei Vorliegen sog. „red flags“ unmittelbar spezifische weitere Abklärungen (z. B. Röntgen, MRT und Labor) und Therapien zur Behandlung der Ursache in die Wege geleitet werden.

Zweitens ist frühzeitig eine Unterscheidung zwischen degenerativen oder entzündlichen Schmerzen vorzunehmen, um im letzteren Fall eine rheumatologische Evaluation einzuleiten. Bei degenerativen Rückenschmerzen treten in erster Linie muskuloskeletale und bei Neurokompression neuropathische Schmerzen auf, die sich mit den folgenden Ausstrahlungscharakteristika unterscheiden:

1. lumbovertebrale Schmerzen, die v. a. belastungsabhängig in der Vertikalen auftreten und einer segmentalen Verteilung folgen;
2. lumbospondylogene Schmerzen, die durch ein Ausstrahlen gekennzeichnet sind, deren Ausbreitung einem Dermatom zu entsprechen scheint, diesem denn aber doch nicht komplett entspricht. Diese Schmerzen werden auch als „pseudoradikulär“ oder „referred pain“ beschrieben. Dabei spielen auch muskuläre oder Sehnenstrukturen eine wichtige Rolle;
3. radikuläre neuropathische Schmerzen mit Schmerzausbreitung entlang eines definierten Dermatoms.

Drittens sind im Hinblick auf die Risiken einer Chronifizierung folgende Kofaktoren zu eruieren: psychosoziale Faktoren, Leidensdruck, schmerzbedingte Beeinträchtigung im Alltag und Arbeitsleben, temporales Verlaufsmuster und Auftreten. Sind solche Faktoren vorhanden, ist in der Regel direkt auch ein multiprofessionelles Assessment sinnvoll.

## Pathogenese chronischer Rückenschmerzen

Chronische Rückenschmerzen werden in ungefähr 90 % der Fälle als unspezifisch dargestellt, d. h. sie können nicht durch Läsion einer Körperstruktur erklärt werden [23]. Der Begriff „unspezifisch“ ist allerdings umstritten, da keine eindeutige Trennung zwischen spezifischen und unspezifischen Schmerzen möglich ist. Unspezifische Rückenschmerzen stellen somit keinen homogenen Symptomkomplex dar, sondern beinhalten heterogene biopsychosoziale Beeinträchtigungen und Schmerzverhaltensmuster. Daher sollten neben Ärzten/Ärztinnen auch andere Gesundheitsprofessionen wie Physiotherapeut:innen, Psycholog:innen, Ergotherapeut:innen und Sozialarbeiter:innen in Anamnese und Behandlung involviert werden [4, 10].

Chronische Rückenschmerzen werden im neuen ICD-11 den primären und sekundären muskuloskeletalen Schmerzen zugeordnet [23]. Am häufigsten entwickeln sich Rückenschmerzen in Verbindung mit degenerativen Veränderungen der Wirbelsäule. Dabei ist die Ätiologie meist multifaktoriell, und das Schmerzausmaß korreliert selten mit dem Ausmaß der Veränderungen. Diskale Degeneration, Osteochondrose, Neurokompression, Facettengelenkdegeneration und myofasziale Reizung (Verspannung, Triggerpunkte) können Ursachen von tieflumbalen Rückenschmerzen sein, aber auch keine Schmerzen verursachen [12].

## Chronifizierung von Rückenschmerzen

Verschiedene Faktoren beeinflussen das Risiko der Progressionen von akuten in chronische Rückenschmerzen. Diese sind genetische Faktoren, das weibliche Geschlecht, psychosoziale Faktoren (d. h. „yellow flags“ z. B. sedentärer Lebensstil, Katastrophisieren, Angst und Stress) und psychisch traumatische Verletzungen. Außerdem beeinflussen berufliche Risikofaktoren (d. h. „blue flags“, z. B. schwere körperliche Arbeit, niedrige Arbeitszufriedenheit) die Chronifizierung. Diese Risikofaktoren scheinen auf verschiedene Art dazu beizutragen, dass sich bei einer Chronifizierung der Schmerzen auch anatomische und funktionelle Veränderungen des zentralen Nervensystems objektivieren lassen [17] und unter Therapie reversibel sind [21].

Chronische Rückenschmerzen sind Ausdruck komplexer biopsychosozialer Vorgänge, die neben degenerativen Veränderungen auch mit psychosozialen Faktoren (z. B. belastendende berufliche Situation) [7], hohem Leidensdruck [8] und schmerzbedingten Beeinträchtigungen im Alltag (z. B. Arbeit, Aktivitäten des täglichen Lebens) [22] einhergehen und unterschiedliche temporale Verlaufsmuster zeigen.

Chronische Rückenschmerzen sind Ausdruck komplexer biopsychosozialer Vorgänge

**Figure 1:**
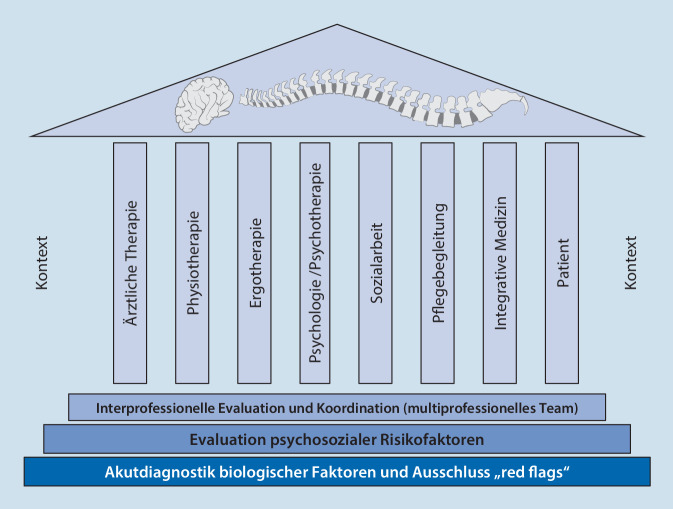

Daher empfehlen Leitlinien nicht nur die Evaluation psychosozialer Risikofaktoren, sondern auch einen multiprofessionellen Behandlungsansatz [2, 4, 10]. Die Behandlung der Rückenschmerzen sollte auf 8 Säulen stehen, wobei das Dach die noziplastischen Schmerzen darstellen [6]. Unter Berücksichtigung des biopsychosozialen Schmerzmodells sollten die verschieden Disziplinen den Patienten simultan stützen und von einem zentralen Koordinator vereint werden (Abb. 1). Das Säulenmodell zeigt, dass nicht nur die verschiedenen Gesundheitsprofessionen, sondern auch der Patient mit seinem Umfeld eine tragende Säule im multiprofessionellen Team sein soll.

Daher sollte für das Gesamtkonzept der Behandlung auch eher der Begriff interprofessionell verwendet werden. Sobald verschiedene ärztliche Disziplinen mit involviert sind, kann auch von einer interdisziplinären und interprofessionellen Schmerztherapie gesprochen werden. Für die Indikationsstellung eines solchen Konzeptes eignet sich besonders eine interdisziplinäre und interprofessionelle Schmerzsprechstunde mit Einbezug der Patienten oder ein entsprechendes Schmerzboard.

## Multiprofessionelle Therapieansätze

Multiprofessionelle Therapieansätze sind derzeit der State-of-the-Art in der Behandlung von chronischen Rückenschmerzen und zeigen sich als wirksamer, aber auch kostenintensiver als Monotherapien [3, 4, 10, 15]. Zwei Cochrane-Metaanalysen zeigten, dass multiprofessionelle Rehabilitationsprogramme gegenüber Monotherapien überlegen waren. So hatten Patienten unter multiprofessioneller Behandlung signifikant weniger Schmerzen und einen geringeren Behinderungsgrad, weniger Fehltage und eine höhere Wahrscheinlichkeit, innerhalb von 12 Monaten an den angestammten Arbeitsplatz zurückzukehren [10, 15]. Eine weitere Metaanalyse aus 47 randomisierten kontrollierten Studien ergab, dass die Länge, Intensität und Kontaktfrequenz der multimodalen Therapie keinen Einfluss auf den gesamten Therapieerfolg (Schmerz, Behinderung, Arbeitsstatus, Lebensqualität, Depression und Angst) hatte. Hierbei zeigte sich in der Subanalyse ein positiver Effekt von längeren Therapien (> 5 Wochen) auf Schmerz und Behinderung [3]. Elbers et al. zeigten in einer Metaanalyse von 72 Kohortenstudien, dass multiprofessionelle Therapieansätze lang anhaltend wirksam in Bezug auf Lebensqualität und Wohlbefinden für Patienten mit chronischen Schmerzen sind, die Therapieansätze jedoch sehr unterschiedlich waren [4].

Multiprofessionelle Rehabilitationsprogramme sind Monotherapien überlegen

Multiprofessionelle Therapieansätze fokussieren nicht nur auf die Einschränkungen in den Körperfunktionen, sondern auch auf die Beeinträchtigung in den Bereichen Aktivität und Partizipation. Dabei stehen gleichberechtigt neben den ärztlichen Interventionen mit Pharmakotherapie, interventioneller Schmerztherapie und Verordnungen weiterer Therapien auch die Angebote der anderen Professionen aus Physiotherapie, Ergotherapie, Pflege, Psychologie und klinischer sozialer Arbeit [3, 4, 10, 15]. Das Besondere hierbei ist, dass die verschiedenen Professionsangehörigen ihre Interventionen nicht parallel anbieten, sondern überlappend und diese regelmäßig aufeinander und gemäß individuellem Patientenbedarf abstimmen.

### Ärztliche pharmakologische Therapie

Das Ziel der Pharmakotherapie sind Verabreichung und Einstellung von Analgetika als wichtige Behandlungsstrategie. Aufgrund der möglichen Nebenwirkungen, Kosten und der Gefahr einer Fehlbelastung unter ausgeschalteter Nozizeption sollten sie jedoch, wenn immer möglich, ärztlich verordnet und nicht als Dauertherapie verwendet werden. Sie können jedoch temporär einen wichtigen Beitrag leisten, den Circulus vitiosus aus Schmerz-Verkrampfung-Schmerz aufzulösen und dadurch den Patienten für die aktiven Therapien vorzubereiten. Gemäß dem American College of Physicians sind nichtsteroidale Antirheumatika (NSARs) immer noch die Therapie der ersten Linie, während Opioide und Duloxetin als Zweitlinientherapie geführt werden [19]. Ein Cochrane-Review über 15 Studien ergab zwar Hinweise für einen kurzfristigen Effekt von Opioiden bei Patienten mit chronischen lumbalen Rückenschmerzen. Auf Grund der unklaren Langzeiteffekte mit doch erheblichem Abhängigkeitspotential, sollten diese nur in ärztlich gut begleiteten Ausnahmesituaitonen eingesetzt werden. Größere Cochrane-Analysen zu anderen Medikamentenklassen sind noch ausstehend. Auch wenn Benzodiazepine hinsichtlich Schmerz und Gesamteffektivität kurzfristig effektiv zu sein scheinen [24], ist aufgrund der hohen Rate unerwünschter Nebenwirkungen (Schwindel, Schläfrigkeit) Vorsicht geboten [24].

### Ärztliche interventionelle Schmerztherapie

Ähnlich wie die Pharmakotherapie kann auch interventionelle Schmerztherapie an der Wirbelsäule eine therapiebereitende Intention haben. Dabei kann ein „schmerzfreies Fenster“ genutzt werden, eine aktive Stärkung der Rumpfmuskulatur voranzutreiben. Zudem dienen schmerztherapeutische Interventionen auch für differenzialdiagnostische Abklärungen, ob ein Schmerz hauptursächlich von einem Facettengelenk (intraartikuläre Infiltration oder Nervenblockade der Facettengelenke und Iliosakralgelenke [ISG]) oder durch Nervenwurzelirritation (epidurale Nervenwurzelblockade) hervorgerufen wird. Dabei kann für eine verlängerte Wirkung von mehreren Monaten auch therapeutisch die Applikation von Kortison genutzt werden oder, bei eindeutiger Diagnostik, auch durch Thermoablation der Facettennerven ein langanhaltender Effekt erreicht werden.

Diese wirbelsäulennahen Interventionen sollten durch entsprechend ausgebildete Ärzte mit einem Fähigkeitsausweis für interventionelle Schmerztherapie unter Bildgebung mittels Röntgen, Computertomographie (CT) oder Ultraschall erfolgen.

Exemplarisch hierfür zeigt eine Metaanalyse zu epiduraler Steroidinfiltration vs. Placeboinfiltration bei Patienten mit lumbosakralen radikulären Schmerzen allerdings nur einen leichten Vorteil für die Intervention bezüglich Schmerzreduktion und Behinderungsgrad mit lediglich kurzweiligen Effekten (< 3 Monate) [18].

Bei muskulären Schmerzen können auch weniger invasive wiederholte Triggerpunktinfiltrationen oder „dry needling“ eine gute Schmerzreduktion erreichen.

Eine weitere Therapiemöglichkeit bei chronischen, therapierefraktären Schmerzen bietet die Implantation von Neurostimulatoren. Auch wenn der Wirkmechanismus schlecht verstanden ist, wird angenommen, dass sowohl die Transmission von Schmerzsignalen zum Kortex als auch eine Inhibition sympathischer Signale Schmerzsignale unterdrücken. Eine Cochrane-Metaanalyse der vorhandenen randomisierten Studien hierzu ist noch ausstehend.

### Physiotherapie

Die Ziele der Physiotherapie im multiprofessionellen Setting sind die Stärkung der paravertebralen Rumpfmuskulatur, um eine Stabilisierung der Wirbelsäulensegmente zu erreichen, die Stärkung der ventralen Bauchmuskulatur, Verbesserung des Körpergefühls, des Körperbewusstseins und der allgemeinen Fitness, Gewichtsabnahmeeffekte, Rückenschule, Erlernen von Heimübungen und Information über Schmerz. Die Wirksamkeit der Physiotherapie bei Patienten mit chronischen Rückenschmerzen wurde bereits in vielen Studien überprüft [3, 4, 10, 15].

### Ergotherapie

Ziel der Ergotherapie ist die Ermöglichung der Ausführung von Alltagsaktivitäten mit dem Schmerz. Dafür werden Aktivitäten und die Umwelt adaptiert, auf eine Balance zwischen energiezehrenden und anstrengenden Aktivitäten wird geachtet, und Abklärungen und Interventionen am Arbeitsplatz werden durchgeführt [4, 10, 13]. Die Ergebnisse eines systematischen Reviews zeigten, dass die Interventionen am Arbeitsplatz sehr unterschiedlich waren und jene, die auf Adaptationen der Umwelt und Training spezifischer Aktivitäten abzielten sich als wirksam erwiesen [20].

### Psychologie und Psychotherapie

Chronischer Schmerz stellt nicht nur ein somatisches, sondern immer auch ein emotionales Erleben dar. Dabei sind Angst, Traurigkeit, Sehen-heißt-glauben-Paradigma und Minderwertigkeitsgefühl besonders häufig mit Schmerz assoziiert. Die Ziele der Psychologie sind daher die Förderung der Akzeptanz von erlebten Einschränkungen durch den Schmerz, die Verringerung von Stress, Depressivität und Ängsten und die Steigerung der Lebensqualität [11]. Dafür kommen verschiedene Interventionen zum Einsatz wie Patientenedukation, Schmerzmanagementstrategien, und kognitiv-verhaltenstherapeutische Techniken [1].

### Pflege

Die Aufgaben der Pflege im multiprofessionellen Behandlungsteam sind im Rahmen der Patientenbegleitung bei stationären wie auch zunehmend bei ambulanten Patienten unter anderem in der Patientenedukation, Förderung des Schlafs, Unterstützung bei Schleimhauttrockenheit, Obstipation und beim Medikamentenmanagement zu sehen. Hierfür gibt es verschiedene Zertifizierungen und Ausbildungen zu Pain Nurses.

### Integrative Medizin

In der integrativen Medizin werden alle evidenzbasierten therapeutischen Ansätze aus der Komplementärmedizin berücksichtigt und in einen ganzheitlichen Ansatz mit schulmedizinischen Methoden integriert. Hierzu gehören unter anderem Akupunktur, Hypnose, Achtsamkeit, verschiedene Entspannungstechniken und Unterstützung für eine gesunde Lebensweise aus der Mind-Body-Medizin. Auf Basis neuester wissenschaftlicher Erkenntnisse wird dabei empfohlen, Placeboeffekte maximal zu nutzen und Noceboeffekte zu meiden. Dieser Bereich kann von allen beteiligten Professionen genutzt werden.

### Klinische Sozialarbeit

Ziel der klinischen Sozialarbeit ist die Förderung der sozialen beruflichen, familiären und gesellschaftlichen Teilhabe (Qualifikationskonzept Gesundheitsbezogene Soziale Arbeit – QGSA der Deutschen Vereinigung für Soziale Arbeit im Gesundheitswesen [DVSG]). Die Interventionen im multiprofessionellen Kontext umfassen die Abklärung und Beantwortung sozialrechtlicher und sozialversicherungsrechtlicher Fragen, Einleiten von beruflichen Maßnahmen (z. B. Anmeldung bei der Invaliden Versicherung und Abklärung von Umschulung), Abklärung subsidiärer Finanzierung und Vernetzung mit Beratungsstellen [7].

### Patient mit Umfeld

Nicht nur der Patient mit seinem Schmerzerleben selbst steht im Zentrum des multiprofessionellen Ansatzes, sondern auch sein Kontext mit persönlichen Faktoren (z. B. Optimismus) und Umweltfaktoren (z. B. Unterstützung durch Partner, Familie und Arbeitgeber) für die Reintegration am Arbeitsplatz. Es ist Aufgabe des multiprofessionellen Teams, diese Ressourcen zu erkennen und nach Möglichkeit in den Therapieansatz einzubinden.

## Fazit für die Praxis

- Multiprofessionelle Therapieansätze erweisen sich für Patienten mit chronischen Schmerzen als wirksamer als monotherapeutische Ansätze.
- Fokus der multiprofessionellen Zusammenarbeit liegt nicht nur in der Reduktion von Einschränkungen im Bereich der Körperfunktionen und -strukturen (z. B. Schmerz, Bewegungseinschränkung), sondern auf der Ermöglichung von Aktivität und Partizipation in verschiedenen Lebensbereichen (z. B. Arbeit und soziales Umfeld) sowie der Aktivierung von Ressourcen im Umfeld des Patienten.
- Wir plädieren für ein biopsychosoziales Säulenmodell, in dem die Therapie für den Patienten individuell mit den Säulen ärztlicher Therapien, der Physio- und Ergotherapie sowie einer psychologischen, pflegerischen und sozialen und der integrativen Begleitung erfolgt. Auch der Patient selbst und seine Kontextfaktoren stellen eine wesentliche Säule in diesem Modell dar.
